# Gastrointestinal Manifestations and Outcomes of COVID-19: A Comprehensive Systematic Review and Meta-analysis

**DOI:** 10.7759/cureus.47028

**Published:** 2023-10-14

**Authors:** Deep Mehta, Raveena Kelkar, Neel Patel, Parth D Trivedi, Sameer Dawoodi, Dhruvan Patel, Dhanshree Solanki, Akbar Hussain, Sanchitha Nagaraj, Azadeh Khayat, Vikramaditya Samala Venkata, Uvesh Mansuri, Urvish K Patel, Henry Sacks, Ashish Atreja

**Affiliations:** 1 Internal Medicine, Capital Health Regional Medical Center, Trenton, USA; 2 Clinical Research, Icahn School of Medicine at Mount Sinai, New York, USA; 3 Internal Medicine, Cleveland Clinic Akron General, Akron, USA; 4 Public Health, Icahn School of Medicine at Mount Sinai, New York, USA; 5 Internal Medicine, Icahn School of Medicine at Mount Sinai, New York, USA; 6 Gastroenterology, State University of New York Downstate Medical Center, New York, USA; 7 Internal Medicine, Yale New Haven Hospital, New Haven, USA; 8 Gastroenterology, Mercy Fitzgerald Hospital, Darby, USA; 9 Gastroenterology, University of Pennsylvania, Philadelphia, USA; 10 Hospital Administration, Rutgers University, New Brunswick, USA; 11 Internal Medicine, Appalachian Regional Healthcare, Hazard, USA; 12 Internal Medicine, Ramaiah Medical College, Banagalore, IND; 13 Pathology and Laboratory Medicine, Brown University, Providence, USA; 14 Internal Medicine, Cheshire Medical Center, Dartmouth-Hitchcock, Keene, USA; 15 Medicine, MedStar Union Memorial Hospital, Baltimore, USA; 16 Public Health and Neurology, Icahn School of Medicine at Mount Sinai, New York, USA; 17 Environmental Medicine and Public Health, Icahn School of Medicine at Mount Sinai, New York, USA; 18 Internal Medicine (Division of Gastroenterology), Icahn School of Medicine at Mount Sinai, New York, USA; 19 Digital Health, University of California Davis Health, Sacramento, USA

**Keywords:** covid-19, gastrointestinal symptoms, outcomes, diarrhea, nausea, vomiting, anorexia, abdominal pain

## Abstract

Introduction

Pulmonary symptoms are the most prominent manifestations of Coronavirus disease 2019 (COVID-19). However, gastrointestinal (GI) symptoms have been reported widely as well. Literature describing the relation of these symptoms with outcomes of COVID-19 patients is limited in terms of sample size, geographic diversity, and the spectrum of GI symptoms included. We aim to evaluate the association of GI symptoms with outcomes of hospitalized COVID-19 patients.

Methods

A systematic review and meta-analysis of observational studies assessing GI symptoms and outcomes in COVID-19 patients were undertaken using Preferred Reporting Items for Systematic Reviews and Meta-Analyses (PRISMA) criteria and the Meta-analysis of Observational Studies in Epidemiology (MOOSE) checklist. Details on outcomes included ICU vs. non-ICU admission, severe vs. non-severe disease, invasive mechanical ventilation (IMV) vs. no-IMV use, oxygen saturation <90% vs. >90%, in-hospital mortality vs. discharged alive and survivors. We obtained the odds ratio (OR), 95% confidence interval (95%CI), and forest plots. Sensitivity analysis was used to analyze publication bias and heterogeneity.

Results

In 35 studies with 7931 confirmed COVID-19 patients, we found that anorexia (pooled OR:2.05; 95%CI: 1.36-3.09, p=0.0006) and abdominal pain (OR 2.80; 95%CI: 1.41-5.54, p=0.003) were associated with a higher risk of poor outcomes and no such association was found for diarrhea (OR 1.04; 95%CI: 0.85-1.26, p=0.71), nausea (OR 0.73; 95%CI: 0.38-1.39, p=0.34) and vomiting (OR 1.24; 95%CI 0.86-1.79, p=0.25).

Conclusion

The meta-analysis concludes that anorexia and abdominal pain are associated with poor outcomes in hospitalized COVID-19 patients, while diarrhea, nausea, and vomiting have no association. Future research should focus on whether detecting GI invasion in conjunction with fecal polymerase chain reaction (PCR) testing can aid in the early triage of high-risk individuals and improve outcomes.

## Introduction and background

The coronavirus disease 2019 (COVID-19) impacted healthcare systems around the world even before it was deemed a pandemic on March 11, 2020 [1]. In terms of total cases and confirmed fatalities, the United States, India, and Brazil are among the nations that have been most severely impacted [2].

COVID-19 exhibits a broad range of clinical presentations, spanning from asymptomatic cases to severe symptomatic illness. Although pulmonary symptoms, including cytokine storm syndrome, are the most prominent features of the disease, it impacts various organ systems [3,4], including the gastrointestinal (GI) system, central nervous system (stroke), hematological system (deep vein thrombosis/ pulmonary embolism) among others [5-8]. GI symptoms such as nausea/vomiting, low appetite, diarrhea, and abdominal pain have been reported widely in pediatric and adult populations. Research has shown that GI symptoms have been observed in approximately 10% of both adult and pediatric patients with COVID-19 [9]. Most studies have predicted the severity of COVID-19 based on biomarkers [10,11] and pre-existing comorbidities [12-14]. Few studies have explored the association between GI symptoms and COVID-19, and those have been limited by small sample size, geographic diversity, and the spectrum of GI symptoms included [10-14].

In this study, we aim to systematically evaluate the association of GI manifestations, including diarrhea, nausea, vomiting, anorexia, and abdominal pain, with outcomes of hospitalized COVID-19 patients.

## Review

Methods

Endpoint

The aim of this study is to evaluate the association between GI manifestations and outcomes of hospitalized COVID-19 patients. Patients were tested with PCR, serology, and symptoms to confirm a diagnosis of infection with COVID-19. Poor outcomes were defined by admission to the intensive care unit (ICU), oxygen saturation (SpO2) <90%, requirement of invasive mechanical ventilation (IMV), severe disease, or in-hospital mortality. We have included studies that included five GI manifestations: diarrhea, nausea, vomiting, anorexia, and abdominal pain.

Search Strategy and Selection Criteria

**Figure 1:**
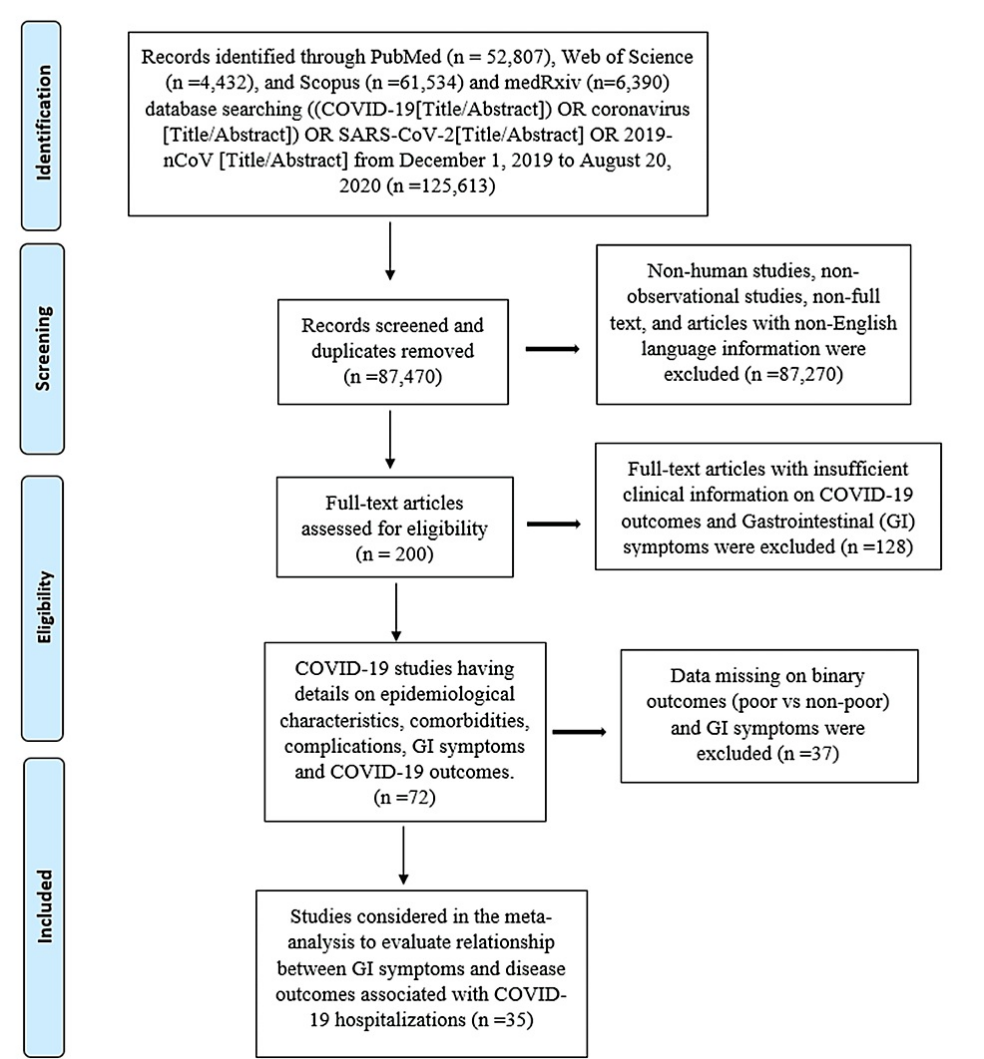
Flow diagram of literature search and study selection process COVID-19: coronavirus disease 2019; SARS-CoV-2: severe acute respiratory syndrome-coronavirus-2

A systematic search was conducted on published studies using Preferred Reporting Items for Systematic Reviews and Meta-Analyses (PRISMA) guidelines [15] and the Meta-analysis of Observational Studies in Epidemiology (MOOSE) checklist [16] from December 1, 2019, to August 20, 2020. We searched PubMed, Web of Science, Scopus, and medRxiv for observational studies that described GI symptoms using the following keyword/Medical Subject Headings (MeSH) terms: ((COVID-19[Title/Abstract]) OR coronavirus [Title/Abstract]) OR SARS-CoV-2[Title/Abstract] OR 2019-nCoV [Title/Abstract]. Studies were included in this meta-analysis if they reported both GI symptoms and outcomes of COVID-19 among hospitalized patients. Non-observational studies, non-English literature, non-full text, and animal studies were excluded. The flow diagram of the literature search and study selection process is described in Figure 1.

Study Selection

All articles were retrieved and reviewed for the availability of data on COVID-19 patients' GI symptoms and outcomes. For quantitative analysis, studies that provided details on outcomes were chosen. DM and RK independently evaluated all identified studies and reviewed entire texts to determine eligibility. Any differences were worked out through discussions with NP and UP.

Data Extraction

We took the following COVID-19-related variables from the included studies: diarrhea, nausea, vomiting, anorexia, stomach discomfort, and COVID-19 results. DM and RK used predetermined data collection forms to gather information on COVID-19 outcomes such as ICU vs. non-ICU admission, severe vs. non-severe disease, IMV vs. no-IMV use, oxygen saturation <90% vs. >90%, in-hospital mortality vs. discharged alive, and survivors. The following individual study details were tabulated: first author's last name, publication month and year, country of origin, sample size, study period, mean or median age, sex, outcomes, and GI manifestations.

Statistical Analysis

Data analysis was performed using Review Manager version 5.3 (The Nordic Cochrane Centre, The Cochrane Collaboration, Copenhagen, Denmark). For studies with more than one outcome comparison, we used data from the most severe outcome to minimize selection bias. The pooled prevalence of GI symptoms was calculated. The Mantel-Haenszel formula with a random-effects model was used to calculate pooled odds ratios (pooled-OR) and their 95% confidence intervals (95% CI) to describe the relationship between GI manifestations and outcomes of COVID-19 patients in each study, and results were represented in the form of forest plots. Each square on the chart area represents an individual study, and the area of each square is equivalent to the weight of the study, which is the inverse of the study variance. The diamond represents the summary measures, and the width corresponds to the 95% CI. The I² statistic was used to assess statistical heterogeneity. Significant heterogeneity was defined as an I² statistic greater than 50%. A p-value of 0.05 or less was regarded as significant. Quality and bias risk were evaluated using the Newcastle-Ottawa Scale (NOS), which measures selection, comparability, and outcome [17]. By eliminating outlying papers from the funnel plot, sensitivity analysis was done to evaluate the impact of heterogeneity. The specific conclusions of the research that were considered for qualitative and quantitative analysis are summarized in the discussion [18-42].

Results

Literature Screening and Characteristics of Included Studies

A total of 200 full-text articles were assessed for eligibility. Of these, 128 articles were excluded due to insufficient clinical information on COVID-19 disease outcomes. Additionally, 37 articles with missing data on outcomes and GI symptoms were also excluded, leaving 35 observational studies comprising 7931 confirmed COVID-19 patients. A flow diagram of studies considered for this meta-analysis is provided in Figure 1. Out of 35 studies, 26 are from China, four from the United States, and one each from Japan, Poland, Korea, Iraq, and Italy. Meta-analysis with random-effects models quantified the study-level impact of GI manifestations on outcomes in hospitalized COVID-19 patients. Most studies had a moderate risk of bias (See Appendix).

The overall prevalence of diarrhea was 13.9% (1071/7714), nausea was 10.9% (252/2302), vomiting was 6.6% (178/2717), anorexia was 30% (801/2670), and abdominal pain was 3.2% (60/1859) in our meta-analysis. These percentages offer a comprehensive understanding of the distribution of symptoms among COVID-19 patients, aiding in better clinical assessment and management. Study-specific characteristics, outcomes, and GI manifestations of individual studies are mentioned in Table 1 [43-77].

**Table 1:** Study characteristics, design, outcomes, and gastrointestinal manifestations ^*^Utilizing the guidelines provided by the American Thoracic Society for community-acquired pneumonia; ^**^Interim guidelines from the WHO and the National Health Commission of China described disease severity and improvement as: Mild cases: mild clinical s/s and absence of pneumonia on imaging, Moderate cases: s/s of fever, respiratory tract symptoms, and visible pneumonia on imaging, Severe cases: Identified by respiratory distress, respiratory rate ≥ 30/min; SpO2 ≤ 93% at rest; and PaO2/FIO2 ≤ 300. Critical/extremely severe cases: Respiratory failure necessitating mechanical ventilation/ICU treatment or experiencing shock; ^***^Patients were categorized as having mild disease (not required HFNC) and severe disease (received HFNC); ^#^Severe disease is an outcome of ARDS, care in ICU, or death. ARDS was diagnosed based on the Berlin definition. COVID-19 was confirmed through real-time RT-PCR assay of nose and/or throat swab samples; ^##^General COVID-19 defined on: (i) notable improvement in respiratory symptoms (e.g., cough, chest discomfort, and shortness of breath) after treatment; (ii) maintenance of normal body temperature for ≥3 days without the use of steroids or antipyretics; (iii) improvement in radiological abnormalities observed in chest CT or X-ray after treatment; (iv) hospital stay of ≤10 days. Cases not meeting these criteria were classified as refractory COVID-19; ^###^Severe cases were identified as patients displaying clinical symptoms of pneumonia (such as dyspnea, tachypnea, SpO2<93%, and need for oxygen therapy). Patients with other symptoms were classified as mild cases. ^¶¶ ^Non-invasive mechanical ventilation encompassed nasal oxygen therapy, mask oxygen inhalation, and HFNC. ARDS: Acute respiratory distress syndrome; COVID-19: coronavirus disease 2019; RT-PCR: reverse transcriptase-polymerase chain reaction; SpO2: peripheral capillary oxygen saturation; PaO2: partial pressure of oxygen; FIO2: fraction of inspired oxygen; HFNC: high-flow nasal cannula

Study	Country	Sample size (n)	Study Period	Mean/Median age (years)	Male (n)	Study design	Outcome	Gastrointestinal manifestations
Huang et al., January 2020 [43]	China	41	December 16, 2019 - January 2, 2020	49	30	Prospective single-center	ICU vs. Non-ICU	Diarrhea
Guan et al., February 2020 [44]	China	1099	December 11, 2019 - January 29, 2020	47	637	Retrospective multi-center	Severe vs. Non-severe*	Diarrhea
Wang et al., February 2020 [45]	China	138	January 1, 2020 - January 28, 2020	56	75	Retrospective single-center	ICU vs. Non-ICU	Diarrhea, Nausea, Vomiting, Anorexia Abdominal pain
Yang et al., February 2020 [46]	China	52	December 24, 2019 - January 26, 2020	59.7	35	Retrospective single-center	Survivor vs. Non-survivor	Vomiting
Zhang et al., February 2020 [47]	China	140	January 16, 2020 - February 3, 2020	57	71	Retrospective single-center	Severe vs Non-severe**	Diarrhea, Nausea, Vomiting, Anorexia Abdominal pain
Chen et al., March 2020 [48]	China	21	December, 2019 - January 27, 2020	56	17	Retrospective single-center	Severe vs. Moderate**	Diarrhea
Mo et al., March 2020 [49]	China	155	January 1, 2019 - February 5, 2020	54	86	Retrospective single-center	General vs. Refractory^##^	Diarrhea, Nausea, Vomiting, Anorexia Abdominal pain
Wang et al., March 2020 [50]	China	69	January 16, 2020 - January 29, 2020	42	32	Retrospective single-center	SpO2<90 vs. SpO2>=90	Diarrhea, Vomiting
Wang et al., March 2020 [51]	China	339	January 1 , 2019 - February 6, 2020	69	166	Retrospective single-center	Survivor vs. Non-survivor	Diarrhea, Nausea, Anorexia
Zhou et al., March 2020 [52]	China	191	December 29, 2019 - January 31, 2020	56	119	Retrospective multi-center cohort	Survivor vs. Non-survivor	Diarrhea
Zheng et al., March 2020 [53]	China	161	January 17, 2020 - February 7, 2020	45	80	Retrospective single-center	Severe vs. Non-severe **	Diarrhea, Nausea
Qin et al., March 2020 [54]	China	452	January 10, 2020 - February 12, 2020	58	235	Retrospective single-center	Severe vs. non-severe**	Diarrhea, Anorexia
Cai et al., April 2020 [55]	China	298	January 11, 2020 - March 6, 2020	47.5	145	Retrospective single-center	Severe vs Non-severe**	Diarrhea
Colaneri et al., April 2020 [56]	Italy	44	February 21, 2020 - February 28, 2020	67.5	28	Retrospective single-center	Severe vs. Mild***	Diarrhea
Goyal et al., April 2020 [57]	USA	393	March 3, 2020 - April 10, 2020	62.2	238	Retrospective multi-center	IMV vs.No IMV	Diarrhea
Nobel et al., April 2020 [58]	USA	278	March 10, 2020 - March 21, 2020	NA	145	Retrospective case-control	ICU vs. Non-ICU	Diarrhea
Tabata et al., April 2020 [59]	Japan	104	February 11, 2020 - February 25, 2020	68	54	Retrospective single-center	Severe vs. Mild^###^	Diarrhea
Zhang et al., April 2020 [60]	China	663	January 11, 2020 - February 6, 2020	55.6	321	Retrospective cohort	Severe and critical vs. Mild and moderate**	Diarrhea, Nausea, Vomiting, Abdominal pain
Nowak et al., May 2020 [61]	Poland	169	March 16, 2020 - April 7, 2020	63.7	87	Retrospective single-center	Survivor vs. Non-survivor	Diarrhea
Huang et. al., May 2020 [62]	China	202	January 22, 2020- February 10, 2020	44	116	Retrospective multi-center	Severe vs. Non-severe**	Diarrhea, Vomiting
Pan et al., May 2020 [63]	China	204	January 18, 2020 - February 28, 2020	52.9	107	Retrospective multi-center	Severe and critical vs. Mild and moderate**	Diarrhea, Vomiting, Anorexia, Abdominal pain
Zheng et al., May 2020 [64]	China	34	January 22, 2020 - Mar 5, 2020	66	23	Retrospective single-center	IMV vs. No IMV^ ¶¶^	Diarrhea
Wang et al., June 2020 [65]	China	344	January 25,2020 - February 25,2020	64	179	Retrospective single-center	Survivor vs. Non-survivor	Diarrhea, Anorexia
Wang et al., June 2020 [66]	China	275	January 20, 2020 - February 10, 2020	49	128	Retrospective single-center	Severe vs non-severe**	Diarrhea
Cao et al., June 2020 [67]	China	80	January 21, 2020 - February 12, 2020	53	38	Retrospective cohort single-center	Severe vs. Non-severe**	Diarrhea, Anorexia
Deng et al., June 2020 [68]	China	65	until April 12, 2020	32.5 vs. 35	36	Retrospective cohort study	(Severe+Critical) vs. Moderate**	Diarrhea, Anorexia
Jang et al., June 2020 [69]	Korea	110	February 19, 2020 - April 15, 2020	56.9	48	retrospective observational study	Severe vs.Non-severe^#^	Diarrhea
Shahriarirad et al., June 2020 [70]	Iran	113	February 20, 2020 - March 20, 2020	53.75	71	Retrospective multi-center	Severe vs Non-severe*	Diarrhea, Nausea, Vomiting, Anorexia, Abdominal pain
Suleyman et al., June 2020 [71]	USA	463	March 9, 2020 - Mar 27, 2020	57.5	204	Retrospective multi-center	Hospitalized vs Discharged home	Diarrhea, Nausea, Vomiting, Anorexia,
Wang et al., July 2020 [72]	China	143	January 15, 2020 - February 28, 2020	58	73	cross-sectional	(Severe and critical) vs.(Mild and moderate)**	Diarrhea, Anorexia
Jiang et al., July 2020 [73]	China	59	February and March, 2020	64	29	Retrospective multi-center	ICU vs. Non-ICU	Diarrhea, Nausea
Li et al., July 2020 [74]	China	548	January 26, 2020 - February 5, 2020	60	279	Ambispective cohort study	Severe vs. Non-severe*	Diarrhea, Vomiting, Abdominal pain
Wei et al., July 2020 [75]	China	276	January 27, 2020 - March 11, 2020	51	155	Retrospective single-center	Severe vs Non-severe**	Diarrhea
Yang et al., August 2020 [76]	China	136	January 28, 2020 - February 12, 2020	56	66	Retrospective multi-center	(Severe+Critical) vs. Mild**	Diarrhea, Anorexia
Ferguson et al., August 2020 [77]	USA	72	March 13, 2020 – May 2, 2020	60.4	38	Retrospective multi-center	ICU vs. Non-ICU	Diarrhea, Nausea, Vomiting
Total		7931						

Diarrhea

**Figure 2:**
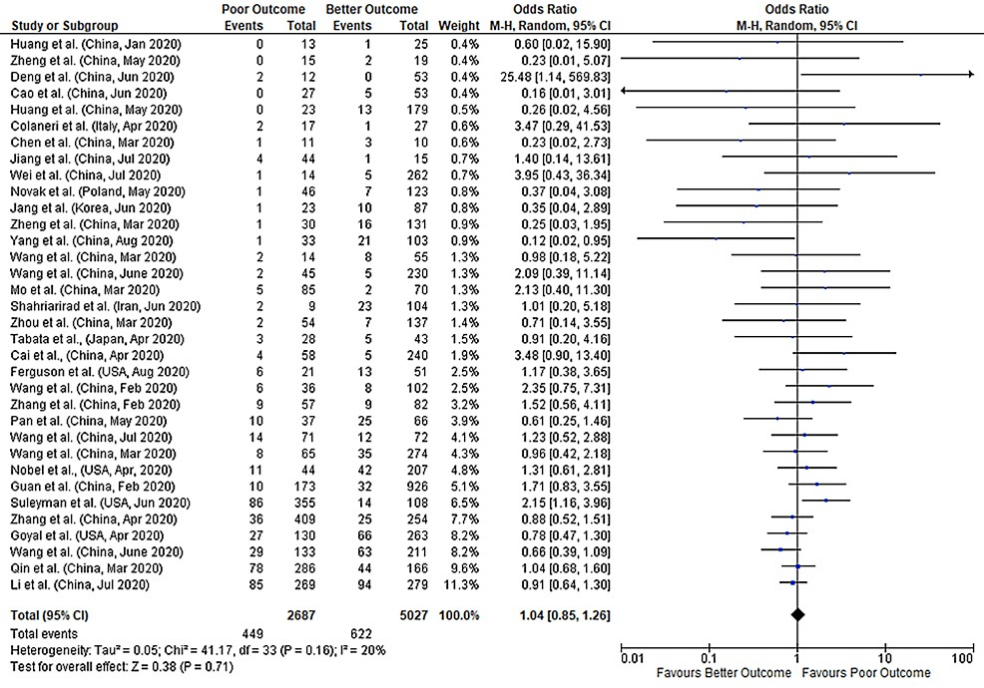
Forest plot showing the association of diarrhea with outcomes in COVID-19 hospitalized patients References: [43-45,47-77] COVID-19: coronavirus disease 2019

A total of 34 studies reported data on both diarrhea and COVID-19 outcomes, comprising data from 7,714 patients. A meta-analysis of these studies found no significant association between diarrhea and COVID-19 outcomes (pooled-OR: 1.04; 95%CI: 0.85-1.26; p=0.71) with 20% between-study heterogeneity (p=0.16) (Figure 2).

Nausea

**Figure 3:**
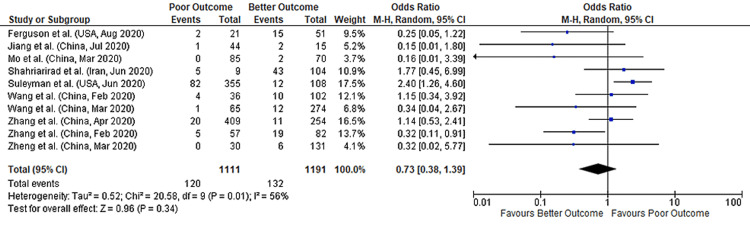
Forest plot showing the association of nausea with outcomes in COVID-19 hospitalized patients References: [45,47,49,51,53,60,70,71,73,77] COVID-19: coronavirus disease 2019

**Figure 4:**
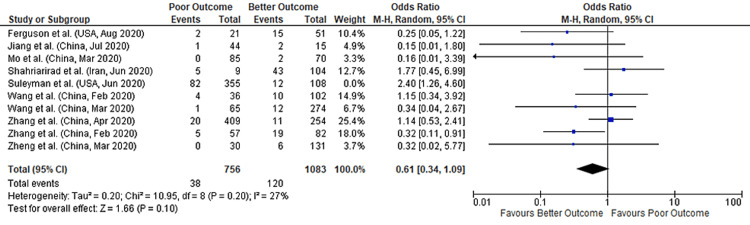
Forest plot showing the association of nausea with outcomes in COVID-19 hospitalized patients (after sensitivity analysis) References: [45,47,49,51,53,60,70,71,73,77] COVID-19: coronavirus disease 2019

**Figure 5:**
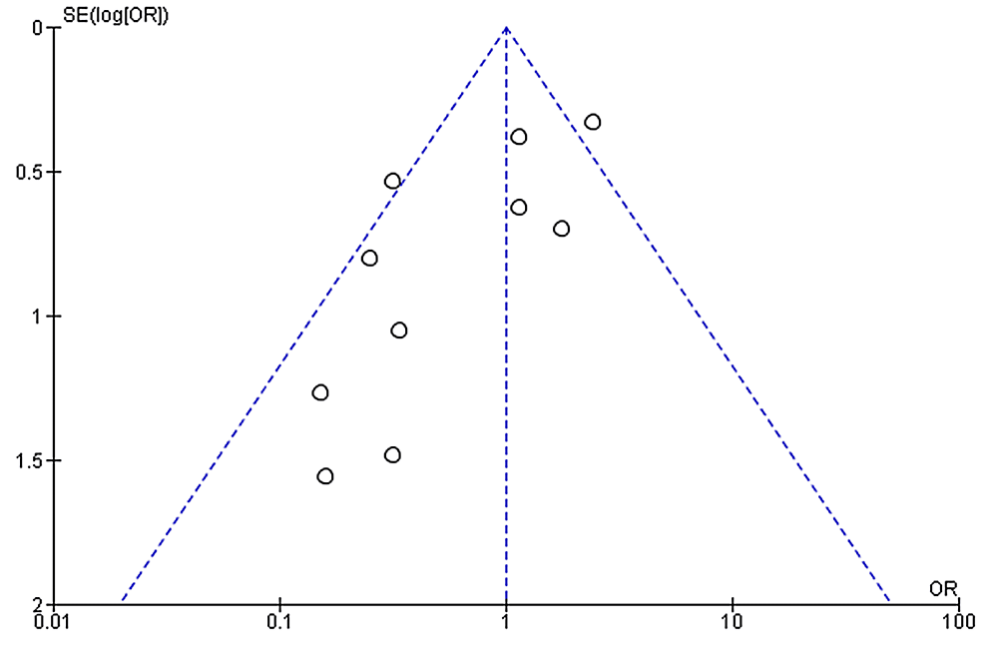
Funnel Plot showing the association of nausea with outcomes in COVID-19 hospitalized patients COVID-19: coronavirus disease 2019

A total of 10 studies reported data on both nausea and COVID-19 outcomes, comprising data from 2,302 patients. A meta-analysis of these studies found no significant association between nausea and COVID-19 outcomes (pooled-OR: 0.73, 95% CI: 0.38-1.39; p=0.34) with 56% between-study heterogeneity (p=0.01) (Figure 3). We performed a sensitivity analysis to account for heterogeneity between the studies. Results after sensitivity analysis also showed no significant association between nausea and COVID-19 outcomes with pooled OR of 0.61 (95%CI: 0.34-1.09; p=0.10) and 27% heterogeneity (p=0.20) (Figure 4). Figure 5 depicts the funnel plot showing the association of nausea with outcomes in COVID-19 hospitalized patients.

Vomiting

**Figure 6:**
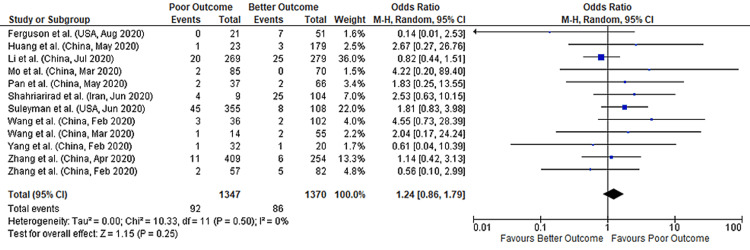
Forest plot showing the association of vomiting with outcomes in COVID-19 hospitalized patients References: [45-47,49-50,60,62-63,70-71,74,77] COVID-19: coronavirus disease 2019

Meta-analysis of 12 studies, including 2717 COVID-19 patients, showed no significant association between vomiting and COVID-19 outcomes (pooled-OR: 1.24; 95%CI: 0.86-1.79; p=0.25), with no heterogeneity (p=0.50; I²=0%) between studies (Figure 6).

Anorexia

**Figure 7:**
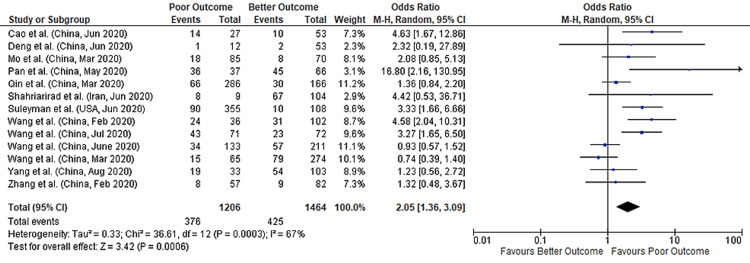
Forest plot showing the association of anorexia with outcomes in COVID-19 hospitalized patients References: [45,47,49,51,54,63,65,67,68,70-72,76] COVID-19: coronavirus disease 2019

**Figure 8:**
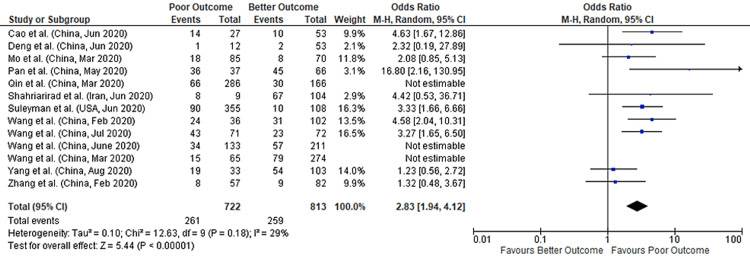
Forest plot showing the association of anorexia with outcomes in COVID-19 hospitalized patients (after sensitivity analysis) References: [45,47,49,51,54,63,65,67,68,70-72,76] COVID-19: coronavirus disease 2019

**Figure 9:**
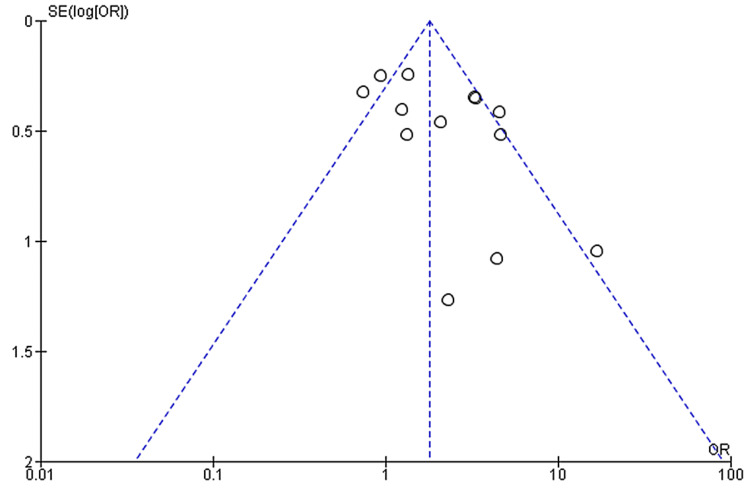
Funnel plot showing the association of anorexia with outcomes in COVID-19 hospitalized patients COVID-19: coronavirus disease 2019

We found 13 studies that provided data concerning anorexia and its association with COVID-19 outcomes. These studies collectively provide a total sample size of 2670 patients for evaluation. Meta-analysis showed that anorexia had a nearly two-fold higher risk of poor outcomes in COVID-19 patients (pooled-OR of 2.05; 95%CI: 1.36-3.09; p=0.0006), with a 67% between-study heterogeneity (p=0.0003) (Figure 7). We performed a sensitivity analysis to account for heterogeneity between the studies. Results after sensitivity analysis also showed a significant association with pooled-OR of 2.83 (95%CI: 1.94-4.12; p<0.00001) with 29% heterogeneity (p=0.18) (Figure 8). Figure 9 depicts the funnel plot showing the association of anorexia with outcomes in COVID-19 hospitalized patients.

Abdominal Pain

**Figure 10:**
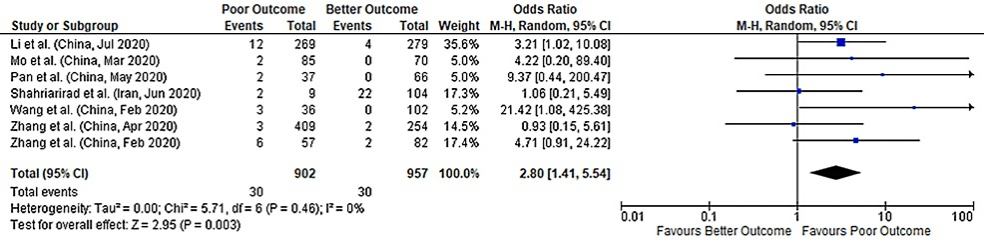
Forest plot showing the association of abdominal pain with outcomes in COVID-19 hospitalized patients References: [45,47,49,60,63,70,74] COVID-19: coronavirus disease 2019

Seven studies provided data on abdominal pain and its association with COVID-19 outcomes with a total sample size of 1859 patients. We found that abdominal pain was associated with a nearly threefold higher risk of poor outcomes in COVID-19 patients (pooled-OR: 2.80; 95%CI: 1.41-5.54; p=0.003) with no heterogeneity between studies (p=0.46; I²=0%). (Figure 10).

Discussion

The global spread of severe acute respiratory syndrome coronavirus 2 (SARS‑CoV‑2) has resulted in the most severe pandemic in a century [18,19]. While vaccines are showing great promise for curbing the spread of this virus [20,21], SARS-CoV-2 may mutate to the point of vaccine resistance. Thus, in addition to developing and improving prophylactic measures, it is imperative to gain a robust understanding of COVID-19, including predictors of COVID-19 poor outcomes [22,23]. In our meta-analysis of 35 studies with 7931 patients who were hospitalized with COVID-19, we found that abdominal pain (OR: 2.80) and loss of appetite (OR: 2.05) were associated with higher odds of poor outcomes. Additionally, we found that diarrhea, nausea, and vomiting were not associated with poor COVID-19 outcomes.

Our findings on predictors of outcomes of COVID-19 are largely concordant with those of a meta-analysis conducted by Mao et al. Their meta-analysis found that among 6,064 patients (January 2020-April 2020), abdominal pain (OR 7.10; 95% CI: 1.93-26.07) was associated with severe disease, while diarrhea (OR 1.22; 95%CI: 0.81-1.84) and nausea/vomiting (OR 1.11; 95%CI: 0.63-1.94) were not. Notably, Mao et al. did not find that loss of appetite was associated with poor outcomes (OR 2.83; 95% CI: 0.92-8.69), in contrast to our findings, though it approached significance (p=0.07) [24]. Kumar et al. noted that patients with abdominal pain had seven times higher odds of having severe illness [25]. Our study showed a narrow confidence interval without overestimating the odds. The meta-analysis by Dorrell et al. (December 2019-May 2020) found that the presence of any GI symptoms was associated with severe COVID-19 disease (OR 2.1; 95% CI: 1.3-3.2), but not associated with mortality (OR 0.9; 95% CI: 0.52-1.54) [26]. Our study builds on these earlier findings and adds to the existing literature by incorporating more recent data and evaluating each GI manifestation separately as a predictor of COVID-19 severity. Another study by Ghimire et al. (December 2019-May 2020) shows that diarrhea is associated with increased severity of COVID-19 disease [27] in contrast to our findings, possibly due to high heterogeneity, lesser number of studies covered, and smaller sample size used to predict severe outcomes of COVID-19. According to a few studies, the prevalence of diarrhea varies from 10% to 16% [28-29], which aligns with our study findings.

The receptor for SARS-CoV-2, the angiotensin-converting enzyme 2 (ACE2) receptor, and the cellular serine protease required for viral entry, transmembrane protease serine 2 (TMPRSS2), are co-expressed in the cells of multiple GI organs, including cholangiocytes, colonocytes, esophageal keratinocytes, ileal absorbing enterocytes, and pancreatic β-cells [30-32]. SARS-CoV-2 mRNA was detected in the stool of 54% of COVID-19 patients [24], and the previously reported point estimate for the prevalence of GI symptoms among patients with COVID-19 was 20% [26]. Collectively, these suggest that direct viral injury is likely the major cause of COVID-19-related GI symptoms [30], though downstream sequelae should also be considered as culprits [33-35]. GI dysfunction as a possible mechanism of neuro-invasion is being widely studied, further highlighting the importance of GI system manifestations [36]. Loss of appetite was the most prevalent GI symptom reported in a recent study [37], similar to our findings. The mechanism of onset of anorexia in patients with COVID-19 remains unclear. Gustatory dysfunction was present in 88% of mild to moderate COVID-19 cases in a recent study [38], which may be one of the contributing factors. The same study also mentioned that olfactory dysfunction was significantly associated with gustatory dysfunction. This may further exacerbate the loss of appetite leading to poor outcomes. We found the prevalence of abdominal pain to be higher in COVID-19 patients with poor outcomes. A recent meta-analysis revealed a link between acute liver injury and elevated liver enzymes (LFT) with severe COVID-19 outcomes [12]. These factors, combined with the direct viral impact on the GI system, could potentially play a role in the occurrence of abdominal pain in these individuals, although the exact causes remain uncertain.

While viral pneumonia is the leading cause of COVID-19-related deaths, it is crucial for both healthcare professionals and the general public to also take into account the non-pulmonary effects and symptoms of the illness. Given that as many as 2% of cases present GI symptoms and no pulmonary symptoms [26], many mild cases may be missed because such patients do not associate their symptoms with COVID-19. Patients presenting without respiratory symptoms are still capable of spreading the infection, as demonstrated by the contribution of asymptomatic and pre-symptomatic transmission to COVID-19 spread [39,40]. Additionally, the isolation of viable SARS-COV-2 from stool samples [41] raises the possibility of fecal-oral transmission [42]. Finally, the emergence of a variant with slightly varied symptomatology cannot be ruled out, and therefore the entire constellation of COVID-19-related symptoms must be considered as we continue to combat this pandemic.

Strengths and Limitations

This study has several notable strengths. Firstly, it includes a relatively large sample size across multiple countries. Additionally, it explores the association between a wider range of GI symptoms and unfavorable outcomes. However, it is important to acknowledge the limitations of this study. All the included studies were retrospective and prospective studies in nature, as randomized trials were lacking, which may introduce an increased risk of publication bias. Moreover, most studies did not specify the timing of outcome occurrence during hospitalization. The heterogeneity among the included studies may be attributed to variations in the definition of disease severity and outcomes. To address this heterogeneity, a sensitivity analysis was conducted. Furthermore, there are additional limitations to consider, including the evolving nature of documenting COVID-19-related GI symptoms, geographic variations in symptom reporting, the predominance of studies from China, and the inability to disaggregate data at the individual patient level. Despite these limitations, our meta-analysis of 7,931 confirmed COVID-19 patients indicates that anorexia and abdominal pain in GI manifestations are relevant in assessing COVID-19 outcomes, while diarrhea, nausea, and vomiting do not serve as predictors of COVID-19 outcomes. Therefore, our study contributes to the existing knowledge by highlighting the utility of GI symptoms in a risk stratification model for predicting severe COVID-19. Given the rapidly increasing number of COVID-19 cases worldwide, waiting for prospective study results would hinder our understanding and clinical management of affected patients.

## Conclusions

The meta-analysis did not find significant associations with diarrhea, nausea, and vomiting, but it identified anorexia and abdominal pain as indicators of poor outcomes in hospitalized COVID-19 patients. The presence of GI symptoms highlights the importance of acknowledging and addressing the non-respiratory aspects of the disease. Future research should delve deeper into the mechanisms linking these symptoms to COVID-19 outcomes and explore potential interventions to mitigate their impact.

## Appendices

**Table 2:** Risk of bias of included studies (NOS) The NOS is a star-based scoring system. It assigns up to a maximum of nine star points for the least risk of bias in three domains: (1) selection of study groups (four points); (2) comparability of groups (two points); and (3) ascertainment of exposure and outcomes (three points) for case-control and cohort studies, respectively. Very Good Quality of Study: 3-4 stars in the selection domain AND 1-2 stars in the comparability domain AND 2-3 stars in the outcome/exposure domain Good Quality of Study: 2 stars in the selection domain AND 1-2 stars in the comparability domain AND 2-3 stars in the outcome/exposure domain Not Good Quality of Study: 0-1 star in the selection domain OR 0 stars in the comparability domain OR 0 or 1 star in the outcome/exposure domain NOS: Newcastle–Ottawa Scale

Study	Newcastle–Ottawa Scale		
	Selection	Comparability	Outcome
Huang et al., Jan 2020 [43]	***	*	*
Guan et al., Feb 2020 [44]	**	*	*
Wang et al., Feb 2020 [45]	***	*	*
Yang et al., Feb 2020 [46]	**	**	**
Zhang et al., Feb 2020 [47]	**	*	*
Chen et al., Mar 2020 [48]	***	*	**
Mo et al., Mar 2020 [49]	**	**	**
Wang et al., Mar 2020 [50]	***	*	*
Wang et al., Mar 2020 [51]	**	**	*
Zhou et al., Mar 2020 [52]	***	*	**
Zheng et al., Mar 2020 [53]	***	*	*
Qin et al., Mar 2020 [54]	**	*	**
Cai et al., Apr 2020 [55]	**	*	*
Colaneri et al., Apr 2020 [56]	***	*	*
Goyal et al., Apr 2020 [57]	***	**	*
Nobel et al., Apr 2020 [58]	**	*	*
Tabata et al., Apr 2020 [59]	***	**	*
Zhang et al., Apr 2020 [60]	***	*	*
Nowak et al., May 2020 [61]	***	*	**
Huang et. al., May 2020 [62]	***	*	**
Pan et al., May, 2020 [63]	**	*	*
Zheng et al., May 2020 [64]	**	*	*
Wang et al., Jun 2020 [65]	**	**	**
Wang et al., Jun 2020 [66]	**	*	*
Cao et al., Jun 2020 [67]	**	*	**
Deng et al., Jun 2020 [68]	***	*	**
Jang et al., Jun 2020 [69]	**	**	**
Shahriarirad et al., Jun 2020 [70]	***	*	**
Suleyman et al., Jun 2020 [71]	**	*	*
Wang et al., Jul 2020 [72]	***	*	**
Jiang et al., Jul 2020 [73]	***	*	**
Li et al., Jul 2020 [74]	***	*	**
Wei et al., Jul 2020 [75]	***	*	**
Yang et al., Aug 2020 [76]	***	**	**
Ferguson et al., Aug 2020 [77]	**	*	**
